# Flower-like Na_2_O nanotip synthesis via femtosecond laser ablation of glass

**DOI:** 10.1186/1556-276X-7-404

**Published:** 2012-07-18

**Authors:** Champika Samarasekera, Bo Tan, Krishnan Venkatakrishnan

**Affiliations:** 1 Department of Aerospace Engineering, Ryerson University, 350 Victoria Street, Toronto, ON, M5B 2K3, Canada; 2 Department of Mechanical and Industrial Engineering, Ryerson University, 350 Victoria Street, Toronto, ON, M5B 2K3, Canada

**Keywords:** Femtosecond laser ablation, Nanostructure, Formation mechanism, Nonmetallic glasses (silicates), Na_2_O, 81 Materials science; 81.07.-b nanoscale materials and structures: fabrication and characterization; 81.16.-c methods of micro- and nanofabrication and processing

## Abstract

The current state-of-the-art in nanotip synthesis relies on techniques that utilize elaborate precursor chemicals, catalysts, or vacuum conditions, and any combination thereof. To realize their ultimate potential, synthesized nanotips require simpler fabrication techniques that allow for control over their final nano-morphology. We present a unique, dry, catalyst-free, and ambient condition method for creating densely clustered, flower-like, sodium oxide (Na_2_O) nanotips with controllable tip widths. Femtosecond laser ablation of a soda-lime glass substrate at a megahertz repetition rate, with nitrogen flow, was employed to generate nanotips with base and head widths as small as 100 and 20 nm respectively, and lengths as long as 10 μm. Control of the nanotip widths was demonstrated via laser dwell time with longer dwell times producing denser clusters of thinner nanotips. Energy dispersive X-ray analysis reveals that nanotip composition is Na_2_O. A new formation mechanism is proposed, involving an electrostatic effect between ionized nitrogen and polar Na_2_O. The synthesized nanotips may potentially be used in antibacterial and hydrogen storage applications.

## Background

Nanotips are a subset of nanostructured materials harboring size-dependent properties that make them uniquely suited to a wide variety of applications in biosensing [1,2], high efficiency solar cells [3,4], and light emission/detection [5,6]. Similarly, soda-lime silicate glasses are among the most ubiquitous of commercial glasses found in windows, containers, bioactive implants [7], and as low cost substrates for thin-film photovoltaics [8]. Previous studies have shown that increasing the Na_2_O content (a major component of soda-lime silicates) of glass can induce a cytotoxic response in cells [9], and randomly oriented nanoscale rod-like structures have demonstrated cell death through reduced cell adhesion [10]. Thus, nanostructured soda-lime silicates may open up new avenues of relevance in antibacterial applications. Additionally, it has been shown that up to 3.0 wt.% hydrogen can be reversibly absorbed by Na_2_O [11]. Improving the performance of such hydrogen storage materials may lay in the enhanced reactivity through increased surface area that the nanostructuring can provide.

Sodium (Na) is frequently used in the process of synthesizing nanostructures and usually constitutes part of a compound nanomaterial in the form of nanowires [12,13] and nanocubes [14]. Nanostructured Na_2_O can be purchased in the form of spherical nanopowders; however, there is no report on the synthesis of Na_2_O nanotips or nanowires. This may be indicative of a reluctance to work with sodium oxide due to its reactivity with aqueous media. However, this need not be a limiting factor. In antibacterial applications where the shape of the nanostructure is believed to cause reduced adhesion of cells, the nano-morphology must be preserved. In the case of Na_2_O, the preservation of its nanostructure and resistivity to aqueous media can be accomplished by depositing a thin film of material (i.e., sputter coating) over the nanostructured surface. The hydrogen storage work of Xu et. al. required no interaction with aqueous media, and the experimenters worked around Na_2_O reactivity by storing samples in a dry box under argon.

Flower-like arrangements of nanoscale structures have previously been synthesized from other metals or metal oxides, such as ZnO [15-18], CuO [19], Cd(OH)_2_[20], Ni(OH)_2_[21], Fe_3_O_4_[22], TiO_2_[23-25], titanate [26], Pt [27,28], Ni [29], and SiO_x_[30]. The bulk of the aforementioned nanostructures were formed using wet techniques such as sol-gel [23,24], hydrothermal [15,16,22,25,26], solvothermal [29], chemical bath [19,21], galvanic displacement [28], electrodeposition [27], or liquid phase pulsed laser ablation (LP-PLA) [20] synthesis. Besides the high cost of raw materials, most wet techniques also require long processing times and/or the use of hazardous organic solutions, which is likely to result in chemically contaminated products. Even the relatively simple LP-PLA method requires careful choices in liquid media and surfactant. A few researchers have used dry-deposition systems such as radio-frequency plasma-assisted magnetrons [17], pulsed laser deposition [18], or chemical vapor deposition (CVD) [30]. These techniques operate under vacuum conditions that can cause added complications in production scale up, and CVD requires the use of catalysts.

In this article we report the synthesis of Na_2_O nanotip clusters using femtosecond laser irradiation of soda-lime glass. Laser irradiation focused inside soda-lime silicates has been suggested to cause the precipitation of Na nanoparticles [31]. However, to the best of our knowledge, this work is the first instance describing the synthesis of flower-like Na_2_O nanotip clusters. The ultrafast laser technique used in this work results in immediate nanotip processing and requires no surfactant, catalyst, nor pressure chamber. The definitive goal for fabricators is the ability to control growth, geometry, and size of the nanostructures. To achieve such bridling, an understanding of a nanostructure's formation mechanism is required. This paper reports on the morphologies of nanotip structures created on soda-lime glass via femtosecond laser ablation, control of nanotip thickness by changing the amount of time over which the laser delivered pulses to the sample, the importance of Na_2_O in nanotip synthesis, and also proposes formation mechanisms based on the observations of nanotip composition and structure using scanning/transmission electron microscopy (S/TEM) and energy dispersive X-ray spectroscopy (EDX).

## Methods

**Figure 1:**
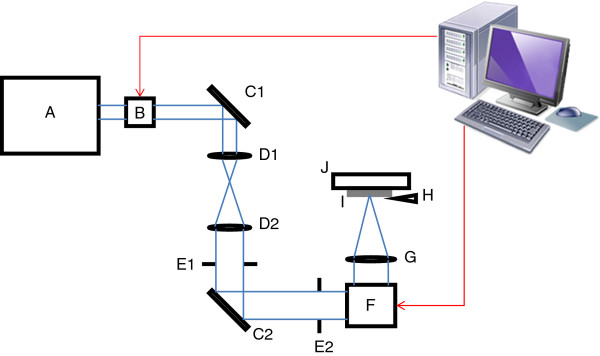
**Schematic diagram of experimental setup.** A, ultrafast laser source; B, acousto-optic modulator; C1 and C2, mirrors; D1 and D2, beam expander; E1 and E2, diaphragms; F, galvoscanner; G, telecentric lens; H, nitrogen nozzle; I, sample; and J, 3-axis stage.

The experimental setup, as illustrated in Figure 1, consists of a 1,030-nm wavelength direct-diode pumped Yb-doped fiber-amplified ultrafast laser source, a beam delivery system, computer controlled galvoscanner, and 3-axis micrometer-resolution translation stage. The laser is capable of delivering pulses with 200 fs duration and pulse repetition rates in the range of 200 kHz to 26 MHz, all at a maximum average power of 15 W. Experiments were performed at repetition rates of 8.4, 12, and 25 MHz and dwell times (time spent delivering laser pulses to a single point on the sample) of 2, 5, and 10 ms. The galvoscanner enabled the samples to be irradiated with a predetermined laser scan pattern, in this case, an array of points with a center-to-center distance of 50 μm. The spot size of the beam at the sample is calculated to be 10.38 μm. All samples were processed under ambient conditions with nitrogen gas flowing at a rate of 10 SCFH over the ablation site. The direction of gas flow was perpendicular to the propagating direction of the incident laser beam.

Glass samples were cut from standard 75 × 25 mm Corning soda-lime glass microscope slides (Ted Pella Inc, Redding, CA) (72% SiO_2_, 15% Na_2_O, 5% CaO, 4% MgO, 2% Al_2_O_3_, 1% K_2_O, and 1% all other constituents). The laser processed samples were first sputter-coated with gold and then examined under SEM. The copper substrate grids were dragged across the surface of ablated samples to collect nanostructures and subsequently observed under TEM. For chemical characterization of the nanostructures, EDX was employed.

## Results

### *Morphology of nanotips*

**Figure 2:**
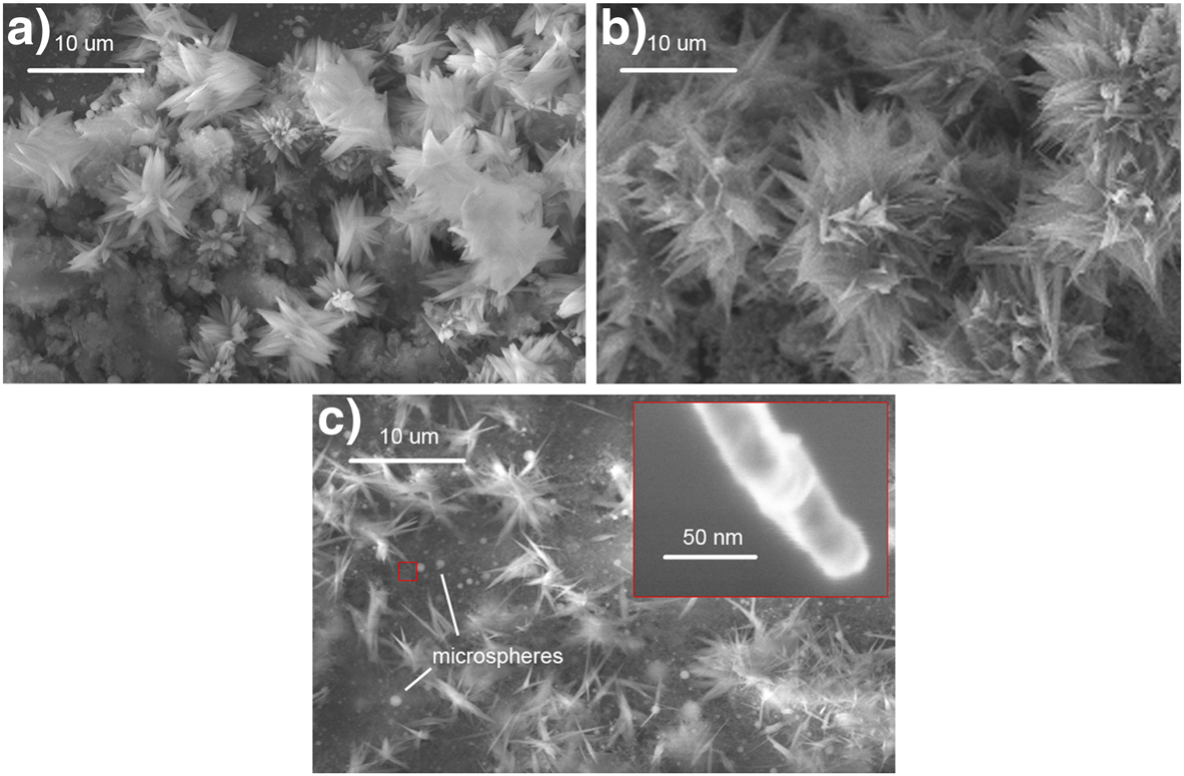
**SEM images of flower-like nanotips formed at a repetition rate of 8.4 MHz and dwell times of (a) 2 ms, (b) 5 ms, and (c) 10 ms.** Inset shows high magnification image of a typical single 10 ms dwell time nanotip with a head width of 20 nm.

All nanotips were randomly oriented and displayed a characteristic-tapered morphology, broad at the base with a distribution of widths from 100 nm to 2 μm and narrowing to a head as small as 20 nm over a length of 1 to 10 μm. Increasing the dwell time at a repetition rate of 8.4 MHz resulted in a greater number of thinner, but not longer, needle-like tips (see Figure 2). The base width of tips at 2 and 5 ms dwell times were typically 1 to 2 μm with more tips per flower generated at 5 ms. When dwell time was increased to 10 ms, base widths shrank between 100 to 500 nm. Interspersed among and around the nanotips were nano and microscale spheres. In some cases, aggregated nanoparticles were also observed.

**Figure 3:**
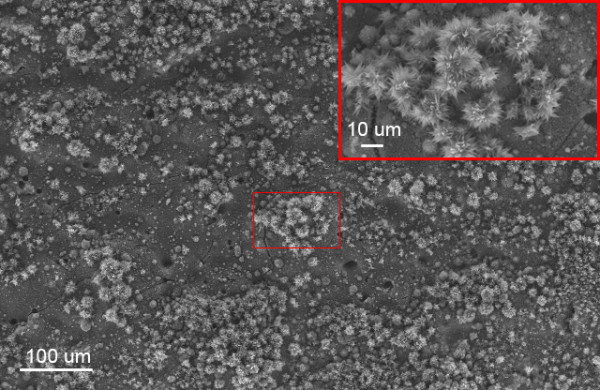
**SEM image of many clusters of flower-like nanotips.** Inset shows higher magnification of a typical cluster.

Also, at this repetition rate nanotip flowers often formed in clusters of 50 μm or larger; however, dramatic differences in nanotip population density (nanotips per unit area) were seen oftentimes within a single ablated region of a sample (see Figure 3).

**Figure 4:**
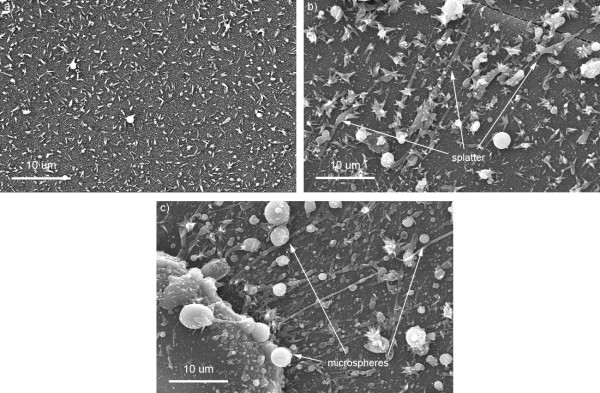
**SEM images of single nanotips formed at a repetition rate of 12 MHz and dwell times(a)2 ms and flower-like nanotips at dwell times of(b)5 ms and(c)10 ms.**

When the repetition rate was increased to 12 MHz (see Figure 4), single nanotips were observed at 2 ms dwell time and at 5 ms dwell time; the formation of flower-like nanotips was more prevalent along with microsphere formation and melt splatter. At 10 ms dwell time, there was further evidence of microsphere formation and melt splatter alongside the continued flower-like nanotip formation. However, at all dwell times clusters of flower-like nanotips as observed at the lower laser repetition rate were not present.

**Figure 5:**
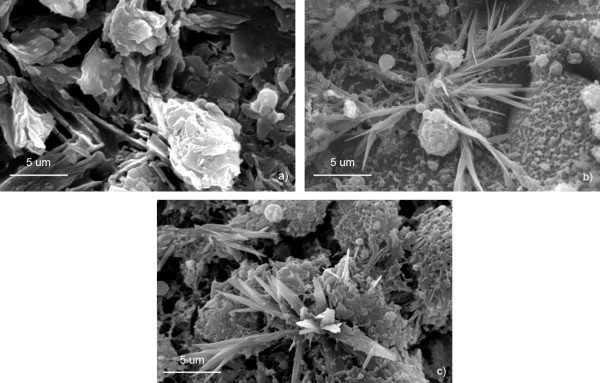
**SEM images of glass substrates after ablation at a repetition rate of 25 MHz and dwell times(a)2 ms, showing no nanotip formation;(b)5 ms; and(c)10 ms showing rare occurrences of flower-like nanotips.**

At repetition rates of 25 MHz, the nanotip formation was typically nonexistent at 2 ms dwell time and sporadic at 5 and 10 ms (see Figure 5).

#### Composition of nanotips

**Figure 6:**
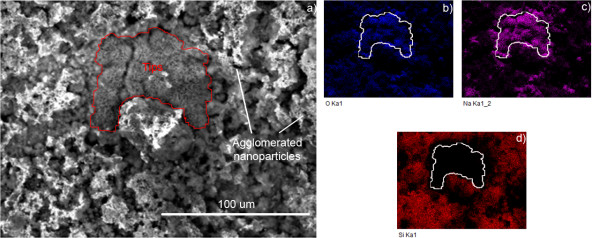
**SEM image of(a)cluster of flower-like nanotips and surrounding nanoparticle agglomerates with corresponding EDX area scans showing high concentrations of(b)oxygen;(c)sodium; and an absence of(d)silicon within the bounded nanotip region.**

Analysis of the flower-like nanotips revealed that the composition of the structures were different from that of the surrounding substrate. Presented in Figure 6 are EDX area scans of a cluster of flower-like nanotips (the region bounded in red on the SEM image and white on the EDX area scans) surrounded by aggregated nanoparticles (the area outside the boundary). This nanotip region shows high concentrations of oxygen and sodium and a clear absence of silicon.

**Figure 7:**
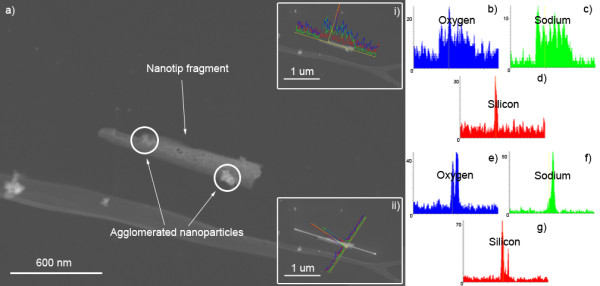
**TEM images** (**a**) **collected nanotip fragment and line scan EDX along i) length, which shows ii) width of fragment.** EDX results along the length and width show (**b**,**e**) oxygen, (**c,f**) sodium throughout the fragment, and (**d,g**) silicon only at the agglomerated particles.

The destructive nature of mounting samples on the substrate grid for use on the TEM resulted in the collection of nanotip fragments. Inspection of individual fragments using line scan EDX revealed that they consisted primarily of oxygen and sodium. Sharp silicon (Si) peaks correspond to the aggregates of Si nanoparticles (see Figure 7). The line scans were purposely drawn well beyond the boundaries of the nanotip so as to compare the element levels of the nanotip to that of the TEM grid. Since the levels of silicon are completely in the noise range except at the two indicated points of interest, we can deduce that silicon is certainly not present throughout the tip.

**Figure 8:**
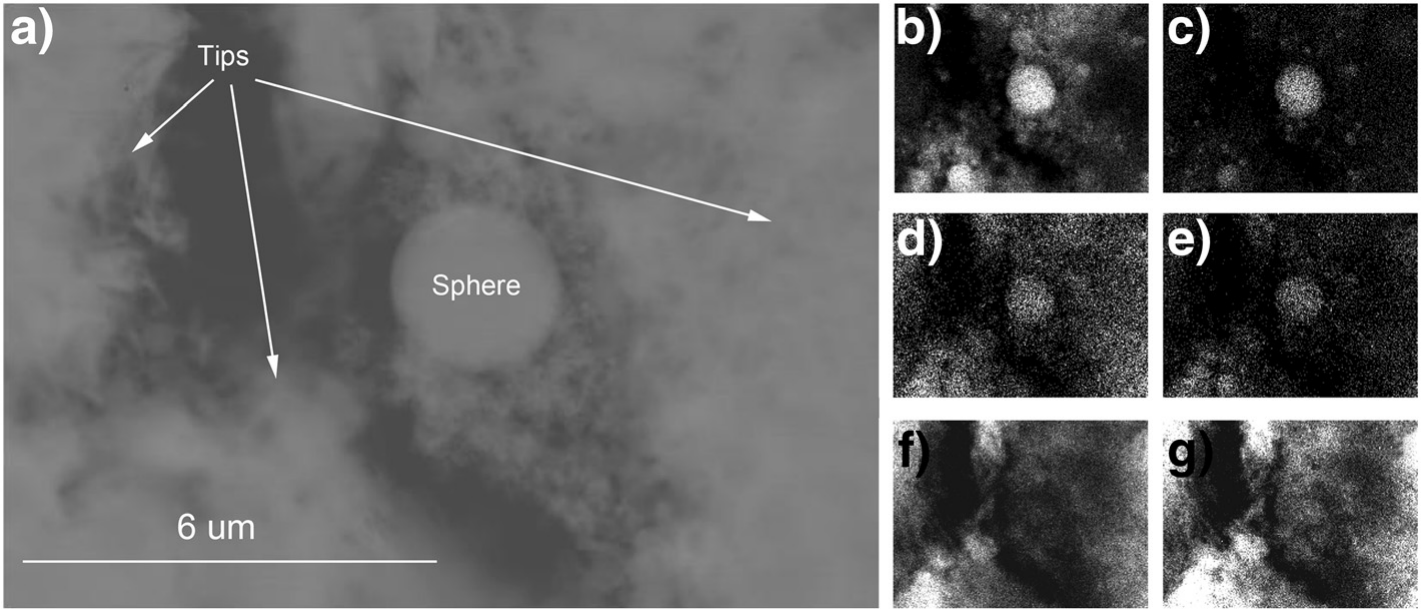
**TEM images(a)nanotip fragments and microspheres with corresponding EDX area scans showing the composition of microspheres to be primarily(b)silicon,(c)calcium,(d)aluminum, and(e)magnesium; while nanotips are composed of(f)sodium and(g)oxygen.**

Collected along with large amounts of nanotip fragments were the microspheres. EDX area scans (see Figure 8) show that the spherical structures differed considerably in composition from their surrounding tips. The spherical particles were primarily constituted of silicon, calcium, aluminum, and magnesium. This was starkly evident in an EDX area scan which showed a dearth of sodium and oxygen in the microsphere.

## Discussion

### Laser interactions

**Figure 9:**
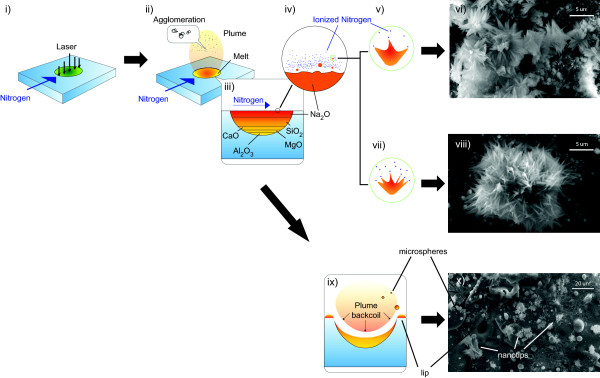
**The proposed formation mechanisms(i) Laser pulses induce optical breakdown; (ii) agglomeration of Si nanoparticles occurs in the plume and a melt layer forms; (iii) melt layer separates soda-lime glass molecules by density; (iv) spallation creates microscale Na**_**2**_**O melt spheres; (v) polar Na**_**2**_**O molecules are electrostatically drawn out towards the laser-ionized nitrogen; (vi) the drawn Na**_**2**_**O cools into flower-like nanotip arrangements (dwell time = 2 ms), (vii) at longer dwell times more nitrogen is ionized, (viii) resulting in thinner nanotips (dwell time = 10 ms); (ix) melt that is now low in Na**_**2**_**O is expelled via plume backcoil pressure to form melt spheres composed of the higher density molecules; (x) typical crater and characteristic lip indicative of a backcoil pressure event (dwell time = 2 ms).**

The presence of several types of structures (single/clustered nanotips, microspheres, and nanoscale aggregates) would appear to indicate multiple formation mechanisms. Much is understood about the material breakdown processes during laser ablation [32]. The optical breakdown of materials is achieved through avalanche ionization of electrons that transfer energy to the lattice of a target material. The energy from a femtosecond laser pulse is deposited into a target faster than the electron-phonon scattering time. Under laser irradiation a solid target will undergo several phenomena simultaneously: direct vaporization of the solid forming a rapidly expanding plasma [33], direct fragmentation of the bulk solid into nanoparticles [34], and material melt forming a thin (few microns thick) molten pool that will explosively boil at high laser intensity and eject microspheres [35]. We shall consider every type of observed structure and see how their formation mechanisms complement each other and fit within the context of laser ablation. Figure 9 illustrates the proposed formation mechanisms together with the experimental evidence. All SEM images were of samples collected at 8 MHz repetition rate, 15 W average power, 10 SCFH nitrogen flow rate, and varying dwell times of 2 and 10 ms.

The optical breakdown of transparent materials occurs when laser pulses of sufficient intensity strike the target substrate's surface (see Figure 9, i) and transfer energy to the electrons via nonlinear ionization, starting the avalanche ionization process. The intensity, *I*, of a laser pulse can be calculated from the well-known equation,

(1) $$I=\frac{4P}{f\tau_{p}d_{0}^{2}\pi }$$

where *P* is the laser power, *f* is the repetition rate, *τ*_p_ is the pulse duration and *d*_0_ is the laser spot diameter. Given the values of the laser parameters used in this experiment, the calculated intensity at the sample surface for repetition rates of 8.4, 12, and 25 MHz are 1.1 × 10^13^, 7.0 × 10^12^, and 3.5 × 10^12^ W·cm^−2^ respectively. The first and second values are well within the threshold intensity for nitrogen ionization (approximately 3.5 × 10^12^ W·cm^−2^) [36]; however, the calculated intensity at a repetition rate of 25 MHz is at the cusp of the ionization threshold. The values calculated here are for an ideal system and of course, further reductions in intensity should be expected from optical losses.

At 8.4 MHz the calculated laser intensity at the focus would ionize nitrogen at a rate of 10^7^ s^−1^[36] but falls as a function of height above the focal plane due to the increased laser spot diameter. Since the beam is Gaussian, its diameter, *d*, will vary along the distance, *z* (also the direction of propagation), according to [37],

(2) $$\text{d}\left(z\right)=d_{0}{\left(1+{\left(\frac{\lambda z}{\pi {\left(\frac{d_{0}}{2}\right)}^{2}}\right)}^{2}\right)}^{1/2}$$

where *d*_0_ is the laser spot diameter at the focal plane and *λ* is the wavelength. At a distance of 10 μm above the focal plane, the beam intensity drops by approximately 10% resulting in a tenfold decrease in the rate of nitrogen ionization. At a distance of 130 μm above the sample surface, the beam diameter expands to 20 μm, and the beam intensity is too low to ionize nitrogen. As shall be explained later, this layer of ionized nitrogen may play a critical role in the formation of nanotips.

### Proposed formation mechanism

The Si formations on nanotip fragments observed under TEM are evidently the result of aggregating nanoparticles and are usually attributed to nucleation and condensation of the vaporized material [34]. In this process, beams of sufficiently high laser fluence heat the region of a target material and vaporize atoms and molecules creating plasma. This plasma will expand as a plume due to continued heating by the laser. The vapor plume begins to cool as it propagates outward interacting with the ambient environment and as a result, the vaporized atoms condense and particles begin to aerosolize (see Figure 9, ii). The particles may then continue to collide with each other forming larger nanoscale aggregates [33].

The microscale spherical particles generated in this study are indicative of formation from a liquid state. Furthermore, the compositional differences between the microspheres and the nanotips in our experimental results point to a phase separation taking place. It is highly likely then, that the molecules separate by density (see Table 1).

**Table 1:** Soda-lime glass composition by weight and corresponding molecular densities at 25°C

**Molecule**	**Density (g·cm**^**−3**^**)**	**Wt.%**
Na_2_O	2.270	14
SiO_2_	2.2 – 2.6	73
CaO	3.3	7
MgO	3.580	4
Al_2_O_3_	4.000	2

Depending on their size, these spherical particles can be attributed to spallation and backcoil pressure (1 to 10 μm) [34,35]. In the first scenario, after a melt layer is created (see Figure 9 iii), the tensile stresses induced by the laser create defects (cavities) along the solid-melt interface; these defects combine causing the ejection of droplets. Backcoil pressure is the result of a quickly expanding vapor plume, pushing liquid melt out from the irradiated spot. If the momentum of the melt is higher than the surface tension, droplets will be ejected around a formed rim as shown in Figure 9, ix.

Sodium oxide is the least dense compound in soda-lime glass. Therefore, regardless of the melt ejection process, we can expect sodium oxide to be at the melt surface (see Figure 9, iv). Furthermore, Na_2_O is a polar molecule. The previously described layer of ionized nitrogen may cause an electrostatic effect that attracts the polar liquid phase Na_2_O (see Figure 9, v). As this liquid is drawn out towards the nitrogen ions, it cools and solidifies into nanotips. The drawing process is evidenced by the tapered structure of the nanotips (see Figure 9, vi). In this experiment the nitrogen gas was provided from one nozzle and in only one direction. As laser dwell time increases, the population of nitrogen ions would grow (see Figure 9, vii). This increase in ions would offer more sources of electrostatic attraction for the polar Na_2_O. Thus, a greater number of tips would be drawn from a droplet of the same volume; the formed cluster would have a higher density of tips, but they would be thinner in width. However, this increased ion density does not affect the length of the nanotips (see Figure 9, viii).

The formation of nanotips into clusters larger than the beam diameter (see Figure 3) suggests that the ionization of nitrogen has an expanded area of effect, which can be accounted for by the constant flow of nitrogen disturbing the population of ions and moving them away from the ablation zone. The fewer observed flower-like nanotips at 12 MHz repetition rates are congruent with a lower quantity of ionized nitrogen due to the lower laser pulse intensity. The observed scarcity of nanotips at a 25 MHz repetition rate can be explained from little to no nitrogen ionizing due to the laser pulse intensity dropping below the ionization threshold of nitrogen.

We have previously shown [38] that without nitrogen flow, similar ablation conditions will not result in nanotip but rather nanofiber formation. Given that the threshold intensity for ionization of air is lower than that of nitrogen [36,39], this may seem surprising. However, the high pressure plume formed during vaporization creates a shockwave that pushes the ambient gas away, creating a vacuum [40,41]. Thus, the ablation zone would be devoid of ionized air. The introduction of nitrogen flow during the ablation process forces a mixing of nitrogen gas into the plume. The nitrogen flow also results in rapid cooling, which would aid in halting the vaporization process and initiating melt ejection processes.

Although silicon dioxide (SiO_2_) is similar in density to Na_2_O, the SiO_2_ molecules are nonpolar and, therefore, unaffected by the electrostatic effect, hence the lack of silicon in the nanotip structure. However, this explanation would not hold true for highly ionic calcium oxide molecules (CaO). The higher density of CaO must impede its ability to be drawn into nanotip structures. The melt that has not solidified into nanotips is likely low in Na_2_O; a delayed melt expulsion process (such as backcoil pressure) would then explain the formation of spherical particles rich in SiO_2_ and higher density molecules (see Figure 9, ix). The crater and rim structures are typically evidence of a backcoil pressure event with microspheres and nanotip clusters around the rim (see Figure 9, x).

## Conclusions

We demonstrate a novel method for synthesis of flower-like nanotips on soda-lime glass via femtosecond laser ablation. Nanotips with head widths as small as 20 nm were obtained. The composition of these nanotips has been investigated and shown to be primarily sodium oxide. From the morphology and composition of the nanotips and their surrounding nano and micro structures, a formation mechanism has been proposed. The process begins with a melting of the glass substrate and separation by density of molecules. This is quickly followed by melt ejection of polar Na_2_O particles that interact electrostatically with the ionized nitrogen, creating the characteristic nanotip shape. Further work is being conducted to determine the cytotoxic potential of these Na_2_O nanotip structures for applications in the controlled localized growth of cells (i.e., patterned growth) and their use as antibacterial surfaces. The determination of the hydrogen absorptivity of these Na_2_O nanotips is also planned for possible energy storage opportunities.

## Competing interests

The authors declare that they have no competing interests.

## Authors’ contributions

CS performed the laser ablation experiments, SEM, TEM and EDX measurements, and developed the proposed formation mechanism. BT and KV supervised analyses of the materials, vetting of the proposed formation mechanism, and preparation of the document. All authors have read and approved the final manuscript.
